# Ultrasound-Responsive Biomimetic Superhydrophobic Drug-Loaded Mesoporous Silica Nanoparticles for Treating Prostate Tumor

**DOI:** 10.3390/pharmaceutics15041155

**Published:** 2023-04-05

**Authors:** Qiaofeng Jin, Dandan Chen, Yishu Song, Tianshu Liu, Wenqu Li, Yihan Chen, Xiaojuan Qin, Li Zhang, Jing Wang, Mingxing Xie

**Affiliations:** 1 Department of Ultrasound Medicine, Union Hospital, Tongji Medical College, Huazhong University of Science and Technology, Wuhan 430022, China; 2 Hubei Province Clinical Research Center for Medical Imaging, Wuhan 430022, China; 3 Hubei Province Key Laboratory of Molecular Imaging, Wuhan 430022, China; 4 Department of Cardiovascular Ultrasound, Zhongnan Hospital of Wuhan University, Wuhan University, Wuhan 430071, China

**Keywords:** ultrasound, mesoporous silica nanoparticle, antivascular therapy, sonodynamic therapy

## Abstract

Interfacial nanobubbles on a superhydrophobic surface can serve as ultrasound cavitation nuclei for continuously promoting sonodynamic therapy, but their poor dispersibility in blood has limited their biomedical application. In this study, we proposed ultrasound-responsive biomimetic superhydrophobic mesoporous silica nanoparticles, modified with red blood cell membrane and loaded with doxorubicin (DOX) (F-MSN-DOX@RBC), for RM-1 tumor sonodynamic therapy. Their mean size and zeta potentials were 232 ± 78.8 nm and −35.57 ± 0.74 mV, respectively. The F-MSN-DOX@RBC accumulation in a tumor was significantly higher than in the control group, and the spleen uptake of F-MSN-DOX@RBC was significantly reduced in comparison to that of the F-MSN-DOX group. Moreover, the cavitation caused by a single dose of F-MSN-DOX@RBC combined with multiple ultrasounds provided continuous sonodynamic therapy. The tumor inhibition rates in the experimental group were 71.5 8 ± 9.54%, which is significantly better than the control group. DHE and CD31 fluorescence staining was used to assess the reactive oxygen species (ROS) generated and the broken tumor vascular system induced by ultrasound. Finally, we can conclude that the combination of anti-vascular therapy, sonodynamic therapy by ROS, and chemotherapy promoted tumor treatment efficacy. The use of red blood cell membrane-modified superhydrophobic silica nanoparticles is a promising strategy in designing ultrasound-responsive nanoparticles to promote drug-release.

## 1. Introduction

One of the major obstacles to effective cancer therapy is the poor accumulation of the drug in tumors due to poor penetration of tumor tissue, a situation often caused by the inhomogeneity of tumor blood vessels and by high fluid pressure in the tumor tissue [1,2]. To promote tumor microenvironment and drug accumulation, current drug delivery strategies are often promoted with physical stimulations such as light [3,4,5], magnetism [6,7], or ultrasound (US) [8,9,10]. It has been shown that US can enhance the preferential accumulation of drug carriers and control drug release in tumors by enhancing blood vessel permeability, or even destroying the vascular system via the cavitation effect with microbubbles [11,12] and phase-shift droplet cavitation nuclei [13,14,15].

Currently, microbubbles and phase-shift droplets are the most common US-responsive drug carriers, and have been used to improve the tumor microenvironment via direct antivascular therapy and deliver their cargo into deep tumor tissues [15,16,17,18]. However, they usually show poor stability, short half-life, and low drug payload. Substantial cavitation initiated from microbubbles, which typically circulate for tens of minutes, is typically sustained for less than 30 s [19]. Therefore, multiple injections are required to sustain cavitation-enhanced drug delivery, exceeding the maximum allowable dose for these agents (0.06 mL kg^−1^) [20,21]. The micron size and short lifetime of these particles may not be sufficient for the drug to exit the blood vessel via the enhanced permeability and retention effect. After US exposure, whether the drug is deposited locally in the tumor tissue or washed out is unclear [22]. US-responsive liposomes [23] and polymer nanoparticles (NPs) [24] are small enough to be transported via the enhanced permeability and retention effect, but are more thermally responsive and require extremely high US power. Therefore, stable NPs that can sustain cavitation efficiently and persistently are receiving increased attention [24,25,26]. 

Recently, it has been found that interfacial nanobubbles (INBs) on a hydrophobic surface have a much longer lifetime (orders of days) than bulk nanobubbles (orders of microseconds), which can decrease the cavitation threshold [27,28]. For example, Kwan et al. have recently developed novel solid–gas nanocups that can sustain cavitation activity for several minutes to address this limitation. However, their NPs are based on polystyrene, which is not biodegradable and unable to carry drugs [29,30]. Jin [31] and Yildirim et al. [25,32,33] demonstrated that air bubbles could be generated from hydrophobic mesoporous silica nanoparticle (MSN) nuclei and serve as contrast agents under the excitation of US. These cavitation bubbles were proved to demonstrate an antivascular therapeutic effect in a similar way to microbubbles [34] and droplets [16,18]. Additionally, we demonstrated that nanobubbles emerging on superhydrophobic polytetrafluoroethylene NPs could sustain inertial cavitation (IC) for much longer than microbubbles and droplets, and produce reactive oxygen species (ROS), which would be appropriate for use as sonosensitizers in sonodynamic therapy [35]. 

However, only a small amount of research has taken advantage of the stability of INBs as gaseous bubble-precursors to develop stable nanoscale US-responsive drug carriers [5]. MSNs have been used as drug carriers for biodegradability [36] and large drug payloads due to their large surface areas and pore volumes [37,38]. Yeh’s group recently developed superhydrophobic MSNs loaded with doxorubicin (DOX) capped with β-cyclodextrin. Using interfacial nanobubbles, which induce continuous cavitation and sustained drug release in a single injection, the anti-vascular, sonodynamic, and chemical therapies are combined on a single platform [39]. 

However, the dispersion of MSNs modified by fluorocarbon is poor in an aqueous environment, and the NPs entering the blood are easily detected as invaders by the innate immune system [40]. They are easily eliminated from circulation by the reticuloendothelial system/mononuclear phagocyte system [41]. In the past two decades, polyethylene glycol (PEG), a hydrophilic polymer, has been widely used in the surface coating of NPs [42]. Although PEG modification can reduce the non-specific adsorption of proteins to a certain extent, some studies have shown that PEGylated NPs induce IgM antibody production, stimulate the complement system, and lead to rapid clearance of subsequently injected NPs [43]. Recently, biomimetic nanoplatforms derived from cell membranes have been widely applied in the biomedical field [44,45]. The natural erythrocyte membrane can directly interact with signal regulatory protein-α expressed by phagocytes to send “do not eat me” signals and inhibit the phagocytosis of NPs by the reticuloendothelial system/mononuclear phagocyte system [46]. Studies have found that erythrocyte membrane-coated NPs circulated long after intravenous injection in mice, and were significantly better than the PEG-modified control group [47,48]. 

Herein, we intended to load the chemotherapy drug DOX into MSNs and wrap them in an erythrocyte membrane to obtain a biomimetic drug delivery system. Combined with HIFU, the cavitation effect of US was utilized to enhance the effect of tumor treatment by destroying tumor blood vessels, killing tumor cells, and reducing the toxic and side effects. 

**Figure 1:**
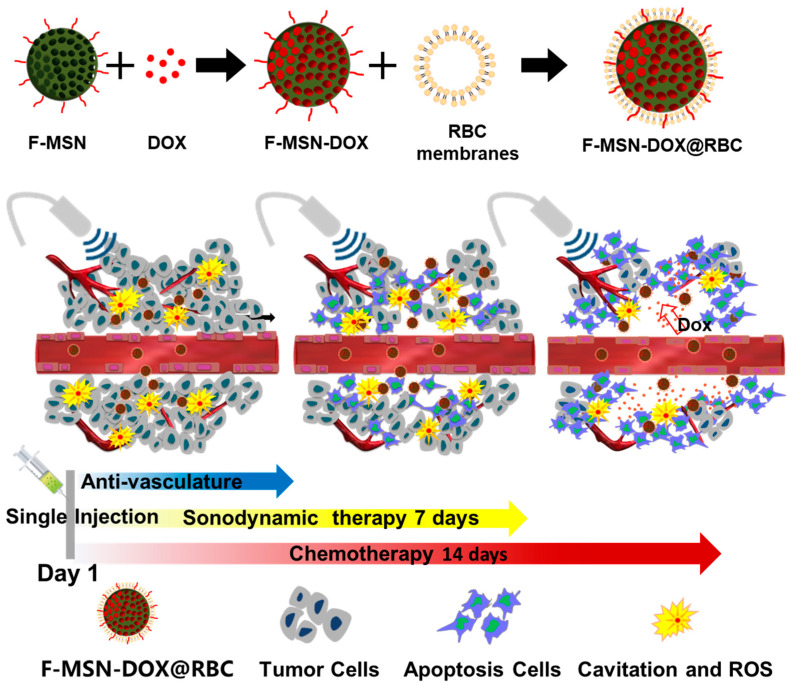
Schematic diagram of the behavior of DOX-loaded superhydrophobic MSN (F-MSN-DOX) with RBC membranes (F-MSN-DOX@RBC). An illustration of the therapeutic process used to treat the tumor by acoustic cavitation and sonodynamic therapy and chemotherapy concurrently using a single dose injection of F-MSN-DOX@RBC.

As shown in Figure 1, a solid superhydrophobic NP, F-MSN, is fully immersed in liquid; the liquid is not directly in contact with the surface of the solid, nanoscopic surface bubbles, or air layer present at the interface. The superhydrophobic NPs can not only be used as efficient cavitation nuclei to enable durable IC under US with a single injection, but also prevent drug leakage during circulation and allow drug release after the gas is consumed by cavitation; thus they may greatly reduce the chemotherapy side-effects in normal tissue. To minimize the possible aggregation of NPs, red blood cell membranes have been used to modify their surface to improve their dispersibility. This study aimed to develop a US-responsive platform to treat solid tumors with US to promote an anti-tumor effect with low chemotherapy side-effects, based on superhydrophobic NPs that can concurrently provide antivascular, sonodynamic, and chemotherapies by acting as an efficient cavitation nucleus.

## 2. Materials and Methods

### 2.1. Materials and Animals

Benzylcetyldimethylammonium chloride, diethylene glycol hexadecyl ether, and tetraethoxysilane were purchased from Sigma Aldrich (St. Louis, MO, USA). Perfluorodecyltriethoxysilane was purchased from yuanye Co., Ltd. (Shanghai, China). DOX was obtained from Aladdin Co., Ltd. (Shanghai, China). DiI (1,1′-dioctadecyl-3,3,3′,3′-tetramethylindocarbocyanine perchlorate) and DiO (3,3′-dioctadecyloxacarbocyanine perchlorate) were purchased from Beyotime Co., Ltd. (Shanghai, China). All solvents and reagents were of analytical or HPLC grade, and all aqueous solutions were prepared using deionized water. For the in vitro cellular level experiments, Dulbecco’s modified Eagle’s medium (DMEM), Roswell Park Memorial Institute (RPMI) 1640, fetal bovine serum (FBS), and phosphate-buffered saline (PBS) were obtained from Gibco (Grand Island, NE, USA).

Male 6–8-week-old C57BL/6 mice (18–20 g) were purchased from the Animal Experimental Center of Tongji Medical College. All the animal experiments were approved by the Institutional Animal Care and Use Committee of Huazhong University of Science and Technology (Wuhan, China) under the Guide for the Care and Use of Laboratory Animals of the National Institutes of Health ([2022] IACUC number 3127).

### 2.2. Synthesis of MSN and F-MSN

A previous method was used to synthesize the parent MSN (MCM-48 type) [39,49]. Briefly, in a polyethylene bottle, 0.592 g of benzylcetyldimethylammonium chloride, 0.208 g of diethylene glycol hexadecyl ether, 17.12 mL of NaOH at 0.4 M, and 460 mL of ultra-pure water were added and stirred overnight at 35 °C. Tetraethoxysilane (4.78 mL) was injected at a rate of 7.5 mL per hour. After aging at 90 °C for 24 h, filtering, washing with water and acetone, and drying at room temperature, the MSNs were collected. Repeated ion exchanges were performed at 35 °C in a dilute HCl–ethanol solution (2% *v/v*) to remove surfactants. In order to remove the adsorbed water, the MSNs (0.1 g) were heated at 150 °C in a vacuum for 12 h. They were then dispersed in a solution containing 1 mL of PFDTS and 10 mL of toluene. The mixture was stirred at 100 °C for 48 h, and the produced superhydrophobic MSNs (F-MSNs) were collected by filtration, repeatedly washed with ethanol, and finally dried at 60 °C for 12 h.

### 2.3. Preparation and Characterization of F-MSN-DOX@RBC

The anticancer drug DOX was loaded into the F-MSNs with the assistance of ethanol. In brief, 2 mL of a 75% ethanol solution was used to dissolve 10 mg of DOX and 10 mg of F-MSN. The ethanol was vaporized at 70 °C three times by replenishing anhydrous ethanol when the liquid phase was almost dry. A centrifuge (10,000× *g*, 5 min) was used to elute excess DOX from the DOX-loaded F-MSNs three times by washing with deionized water [2]. We then prepared vesicles derived from red blood cell (RBC) membranes. In brief, C57BL/6 mice’s orbital blood was collected and stored in anticoagulation tubes. In order to remove the plasma and buffy coat from the blood, 800 g of blood was centrifuged for 5 min at 4 °C. RBCs were then thoroughly washed with 1 mL of ice-cold PBS three times. Then, 0.25 mL of PBS was added for hemolysis in a hypotonic medium for at least 30 min before being cooled in an ice bath. In order to remove the hemoglobin, the released blood was centrifuged at 9000 rpm for 5 min. Two washes with 0.01 M PBS were performed after the pink pellet was collected. A bath sonicator was then used to homogenize the solution, followed by sequential extrusion through 400 nm polycarbonate porous membranes using a mini-extruder (Avanti Polar Lipids, Alabaster, AL, USA). The obtained solution was called RBC membrane-derived vesicles [3]. To coat the RBC membrane onto the surface of the F-MSN-DOX NPs, 2 mL of PBS containing F-MSN-DOX NPs with 1 mg of F-MSNs was mixed with the RBC membrane-derived vesicles derived from 200 µL of whole blood using an intelligent ultrasonic processor (Ningbo Licheng Instrument, Yuyao, China) [4]. The US parameters for this part were as follows: 100 W, on/off = 1/1, 4 min. Then the excess RBC membrane was removed by centrifugation at 10,000× *g* for 5 min, and the resulting RBC-membrane-coated F-MSN-DOX NPs (denoted as F-MSN-DOX@RBC) were stored at 4 °C for further use. 

### 2.4. Characterization of Different NPs

#### 2.4.1. Size Distribution, Zeta Potential and Morphology

The size distribution and concentration of F-MSN, F-MSN-DOX, and F-MSN-DOX@RBC were measured using nanoparticle tracking analysis (NanoSight NS300, Malvern, UK). Zeta-potentials were measured with dynamic light scattering (Zetasizer Nano ZS90, Malvern Instruments, Worcestershire, UK). The morphologies of F-MSN, F-MSN-DOX, and F-MSN-DOX@RBC were evaluated by transmission electron microscopy (TEM, FEI Tecnai G2 F30, Hillsboro, WA, USA) and scanning electron microscopy (SEM, Hitachi SU8010, Tokyo, Japan). In order to measure the contact angle of the NPs, an acetone suspension of NPs (100 mg mL^−1^) was dropped on a slide and evaporated for 0.5 h at room temperature. A contact-angle analyzer (FTA-1000B, First Ten Angstroms) was used to measure static contact angles of the as-prepared NP films.

#### 2.4.2. Nitrogen Physisorption Isotherms

The nitrogen physisorption isotherms of MSN and F-MSN were characterized using a TriStar II3020 specific surface area (Micromeritics, Norcross, GA, USA) at 77 K and a relative pressure (P/P_0_) of 0∼0.99. The samples were degassed under vacuum at 120 °C for 3 h prior to measurement. The specific surface area was calculated using the multi-point Brunauer–Emmett–Teller method in the relative pressure range of 0.05–0.30, and the total pore volume was calculated using Barrett–Joyner–Halenda adsorption data at a relative pressure of 0.95. 

#### 2.4.3. Drug Loading Efficiency

The drug entrapment efficiency (DEE) and drug loading efficiency (DLE) were measured using a multifunctional microplate reader at a wavelength of 480 nm, and they were calculated as follows: DEE = (weight of DOX in F-MSN-DOX/weight of DOX initial added) × 100%, DLE = (weight of DOX_Initial_ − weight of DOX_free_/weight of F-MSN-DOX) × 100%.

#### 2.4.4. In Vitro Drug Release

As previously reported, the in vitro release tests were conducted using a dialysis method and were performed under the sink condition [5,6]. Briefly, 2 mL of a F-MSN-DOX NP dispersion or a F-MSN-DOX@RBC NP dispersion (equal to 1.0 mg of DOX) was added to a dialysis bag (MWCO: 3.5 KDa), and 2 mL of DOX in PBS (500 µg mL^–1^) was used as a control. The dialysis bag was immersed in a conical flask containing 100 mL of PBS, and then the conical flask was shaken at 37 °C and 150 rpm in a constant temperature shaker. At specific time points (1 h, 4 h, 8 h, 12 h, 24 h, 36 h, 48 h, 60 h), 1 mL of the liquid outside the dialysis bag was taken, and 1 mL of fresh PBS solution was added to the conical flask. A multifunctional microplate reader detected the fluorescence values of DOX (480 nm/590 nm) at different concentrations, and the cumulative drug release at each time point was calculated. 

#### 2.4.5. RBC Membrane Characterization

The membrane protein was analyzed using sodium dodecyl sulfate–polyacrylamide gel electrophoresis (SDS-PAGE). A Bicinchoninic (BCA) assay kit was used to measure F-MSN-DOX and F-MSN-DOX@RBC NPs in RBC lysate and RBC vesicles, and measure F-MSN-DOX@RBC NPs in an SDS sample buffer (Invitrogen, Waltham, MA, USA). A 10% SDS–polyacrylamide gel (Beyotime, China) was loaded with 40 g of each sample after they were heated for 5 min at 95 °C. Following 2 h of 120 V running, the polyacrylamide gel was stained with Bromophenol Blue for 3 h and washed repeatedly.

To measure the encapsulation efficiency of RBC vesicles on the F-MSN, DiO and DiI were used to label RBC vesicles (F-MSN@RBC-DiO) and F-MSN (F-MSN-DiI@RBC), respectively, and served as single-labeled groups. Then, RBC vesicles and F-MSN were simultaneously labeled (F-MSN-DiI@RBC-DiO) as double-labeled. Flow cytometry was then used to determine the encapsulation efficiency of RBC vesicles on the F-MSN.

### 2.5. Cell Viability 

Cell viability was assessed under different conditions using the murine prostate cancer cell line RM-1 (American Type Culture Collection, ATCC CRL-3310). A humidified atmosphere with 5% CO_2_ at 37 °C was used for the culture of RM-1 cells. These cells were seeded into 96-well plates (8000 cells/well) and incubated overnight in the culture medium. Five groups were studied: DOX, F-MSN-DOX, F-MSN-DOX@RBC, F-MSN@RBC with US, and F-MSN-DOX@RBC with US. We sonicated US groups under HIFU at a frequency of 2 MHz using 5 MPa and 50-cycle pulses at a power rate of 100 Hz for a period of 5 min. A wash of the cells in PBS, followed by incubation in DMEM containing 10% CCK-8, was conducted after various hours of incubation. Finally, the absorbance of CCK-8 at 450 nm was determined by using a multifunctional microplate reader.

### 2.6. Cell Uptake

Murine macrophages (RAW 264.7) and RM-1 cells were seeded in 6-well plates (10^6^ cells/well) and incubated overnight. Then the cells were treated with F-MSN-DOX or F-MSN-DOX@RBC for 2, 6, 12, and 24 h. We collected cells at preset time points, and the phagocytosis of the different NPs by RAW 264.7 and RM-1 cells was detected by flow cytometry.

### 2.7. In Vivo Distribution

RM-1 cells (5 × 10^6^ cells) were subcutaneously implanted into the right legs of C57BL/6 mice and allowed to grow for about 10 days (tumor volume of 200 mm^3^). Then, a F-MSN-DOX or F-MSN-DOX@RBC solution at an equivalent DOX dose of 5.0 mg (kg BW)^−1^ in 0.1 mL of PBS was injected into the mice via the lateral tail vein (n = 6). After 6, 12, and 24 h injections, the heart, liver, spleen, lung, kidney, and tumor were removed to record ex vivo fluorescence imaging with a small animal imaging system (In-Vivo FX PRO, Bruker, San Jose, CA, USA). 

### 2.8. In Vivo Anti-Tumor Efficacy 

To evaluate anti-tumor efficacy and safety in vivo, an RM-1 xenografted C57BL/6 mouse model was used. The mice were randomly divided into five groups (n = 6 per group) when their tumor volumes reached about 80 mm^3^. F-MSN-DOX or F-MSN-DOX@RBC (1 mg mL^–1^, 150 mg Dox/mouse) was injected intravenously into tumor-bearing mice on day 1, along with 0.15 mL of PBS alone as a control. On days 1, 3, 5, and 7, US groups were sonicated using a 2 MHz HIFU. A HIFU sonication protocol was conducted with a peak-negative pressure of 7 MPa, a cycle time of 5000, and a pulse rate of 18 Hz. In total, the sonication time was about 20–30 min. Mice were monitored every other day to determine the tumor volumes and body weights. Fifteen days after the start of the experiment, the mice were sacrificed. Thereafter, tumors and major organs (e.g., heart, liver, spleen, lung, kidneys) were excised, washed with PBS, and weighed precisely. Further analysis was performed by freezing the tissues at −80 °C after they were fixed in 4% (*w*/*v*) paraformaldehyde buffered by PBS or precooled with liquid nitrogen for 5 min. As determined by Equations (1)–(3), tumor volume, relative tumor volume, and tumor inhibitory rate were calculated.

(1) $$\mathrm{Tumor}\;\mathrm{volume}\;(\mathrm{TV},\;{\mathrm{mm}}^{3})=\frac{L\times S\times S}{2}$$

(2) $$\mathrm{Relative}\;\mathrm{tumor}\;\mathrm{volume}\;(\mathrm{RTV})=\frac{\mathrm{tumor}\;\mathrm{volume}\;\mathrm{of}\;\mathrm{day}\;n}{\mathrm{tumor}\;\mathrm{volume}\;\mathrm{of}\;\mathrm{day}\;1}$$

(3) $$\mathrm{Tumor}\;\mathrm{inhibition}\;\mathrm{rate}\;(\mathrm{TIR},\;\%)=\left(1-\frac{\mathrm{RTV}\;\mathrm{of}\;\mathrm{the}\;\mathrm{experimental}\;\mathrm{group}}{\mathrm{RTV}\;\mathrm{of}\;\mathrm{PBS}\;\mathrm{group}}\right)\times 100\%$$

### 2.9. Statistical Analysis

All data are presented as mean value ± standard deviation (SD) with more than three independent samples, and the standard deviation is shown as an error bar in each graph. Statistical analysis was performed by the Student’s *t*-test or one-way analysis of variance using GraphPad Prism 8.0 (GraphPad Software Inc., San Diego, CA, USA). All the differences were considered to be statistically significant when *p* < 0.05.

## 3. Results

### 3.1. Characterization of F-MSN-DOX@RBC

**Figure 2:**
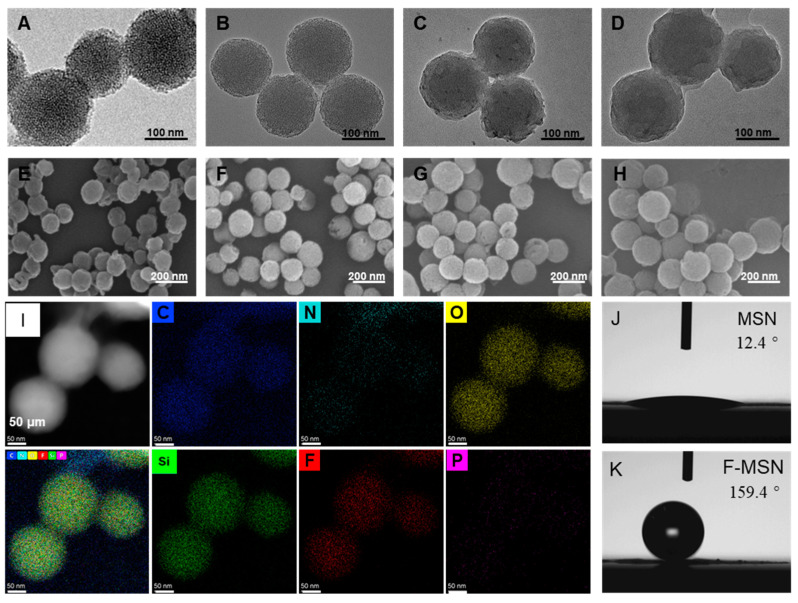
Characterization of NPs. (**A**–**D**) SEM and (**E**–**H**) TEM of the MSN, F-MSN, F-MSN-DOX and F-MSN-DOX@RBC; (**I**) SEM image, energy dispersive X-ray (EDX) elemental mapping of C, N, O, SI, F, and P of F-MSN-DOX@RBC; (**J**,**K**) Contact angle of films made from MSN and F-MSN.

TEM images clearly showed the regular arrangement of mesoporous structures within MSN and the internal structure of the F-MSN, F-MSN-DOX, and the F-MSN-DOX@RBC (Figure 2A–D). The external morphology of the above three NPs was examined by SEM as illustrated in Figure 2E–H, respectively. The energy-dispersive X-ray elemental mapping of F-MSN-DOX@RBC in Figure 2J shows visible elements C, N, O, Si, F, and P on the surface. The contact angle measurement reveals further that fluorocarbon modification has given rise to superhydrophobic NPs, F-MSNs, with a static contact angle of 159.4 degrees. As shown in Table 1, NP tracking analysis technology provided that the mean diameter of the MSNs was 153.7 ± 86.2 nm. After modification of the surface by fluorocarbon chains, slightly larger F-MSNs were obtained with a mean diameter of 171.1 ± 53.9 nm. The larger size of F-MSN-DOX@RBC, with a mean diameter of 232.6 ± 78.8 nm, was attributed to the loading of DOX and the wrapping of the erythrocyte membrane. Changes in the zeta potential of the various NPs also attested to the successful modification at each step (Table 1). MSN featured a type IV nitrogen physisorption isotherm, indicating that the material had a mesoporous structure. Furthermore, the specific surface area, total pore volume, and pore diameter of the MSNs were calculated by the multi-point Brunauer–Emmett–Teller method to be 798.63 m^2^ g^–1^, 0.71 cm^3^ g^–1^, and 3 nm, respectively. 

### 3.2. Drug-Loading, RBC Membrane Envelope Verification, and In Vitro Drug Release

**Figure 3:**
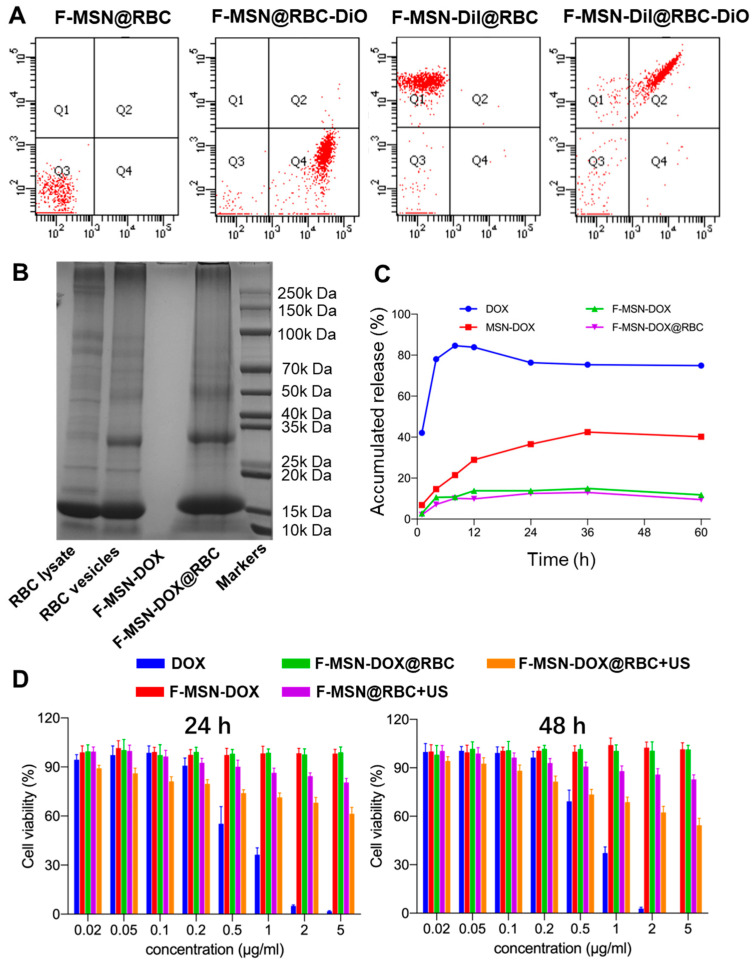
(**A**) The encapsulation of F-MSNs by erythrocyte membrane vesicles assessed by flow cytometry. (**B**) SDS-PAGE of NPs and RBC lysate and RBC vesicles. (**C**) In vitro DOX release profiles of different NPs. (**D**) The viability of RM-1 cells in various groups at 24 and 48 h measured with the CCK-8 assay.

The DEE and DLE of F-MSN-DOX were determined by an indirect method and were 98.54 ± 1.54% and 49.63 ± 0.39%, respectively. Then we used flow cytometry to quantify the encapsulation of F-MSN by erythrocyte membrane vesicles, and the results showed that the encapsulation was about 84.57 ± 3.76% (Figure 3A). SDS-PAGE was carried out to verify the protein expression on the surface of the erythrocyte membrane at different stages, and the results showed that the protein on the surface of F-MSN-DOX@RBC was consistent with that of RBC lysate and RBC vesicles, while no associated protein was measured on the surface of F-MSN-DOX (Figure 3B). According to this result, the expression of the protein on the surface of erythrocytes was not affected by the series of operations during the preparation of F-MSN-DOX@RBC. Using a dialysis method at predetermined times, the release of DOX from different NPs was examined in vitro. In Figure 3C, more than 80% of the free DOX solution was released within 8 h and thereafter began to dissolve. Compared to regular MSNs, F-MSNs effectively reduce drug leakage, and RBC encapsulation did not affect the release of DOX from F-MSN.

### 3.3. Cytotoxicity Assays and Cell Uptake

In this study, the CCK-8 assay was used to measure the viability of RM-1 cells in various groups. As depicted in Figure 3D, under an equivalent DOX concentration and incubation time (i.e., 24, 48, or 72 h), the inhibitory effect of the free DOX group on cell proliferation was stronger than that of the F-MSN and DOX conjugate groups, which could be attributed to free DOX that could easily cross the cell membrane via passive diffusion through a high concentration gradient of the drug. Meanwhile, the F-MSN-DOX@RBC+US group exhibited a more potent inhibitory effect than the F-MSN-DOX group, F-MSN-DOX@RBC group, and F-MSN@RBC+US group. Moreover, the cell viability between the F-MSN-DOX group and F-MSN-DOX@RBC group had no significant difference at different time points (i.e., 24, 48, or 72 h), demonstrating that the F-MSN was effective in preventing drug leakage and could cause targeted drug release only when US was administered.

**Figure 4:**
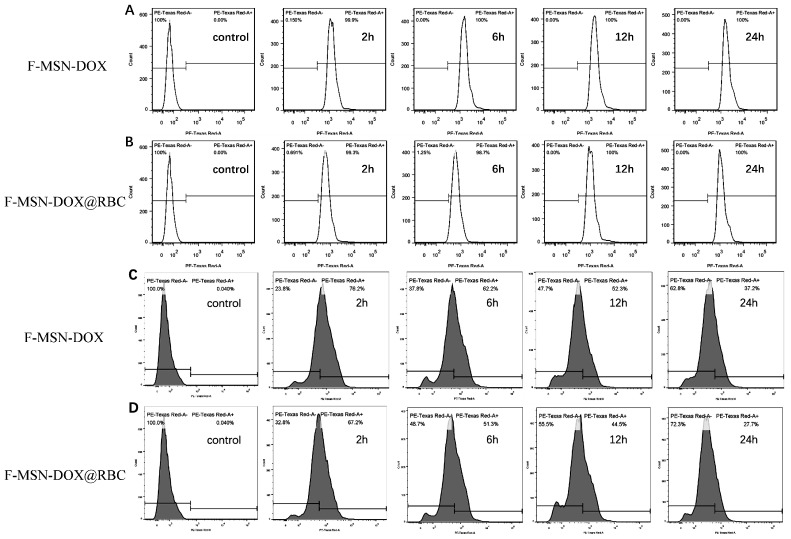
Phagocytosis of nanoparticles. (**A**) F-MSN-DOX and (**B**) F-MSN-DOX@RBC by RM-1 assessed with flow cytometry; (**C**) F-MSN-DOX and (**D**) F-MSN-DOX@RBC assessed by macrophages with flow cytometry.

Then we used flow cytometry to study the phagocytosis of F-MSN-DOX and F-MSN-DOX@RBC NPs by RM-1 tumor cells and RAW 264.7 macrophages. The phagocytosis rates of RM-1 cells for F-MSN-DOX at 2 h, 6 h, 12 h, and 24 h were 99.67 ± 0.49%, 99.20 ± 1.14%, 98.77 ± 1.16%, and 99.70 ± 0.44%, respectively (Figure 4A), and for F-MSN-DOX@RBC were 99.63 ± 0.35%, 99.33 ± 0.65%, 99.17 ± 0.80%, and 99.33 ± 0.65%, respectively (Figure 4B). Comparison of the phagocytosis rates of F-MSN-DOX and F-MSN-DOX@RBC NPs by RM-1 cells at different time points (i.e., 2, 6, 12, or 24 h) revealed no statistical difference (*p* > 0.05), indicating that the erythrocyte membrane coating did not affect the phagocytosis of NPs by RM-1 cells. The phagocytosis rates of RAW 264.7 for F-MSN-DOX at 2 h, 6 h, 12 h, and 24 h were 73.83 ± 2.97%, 63.90 ± 4.40%, 55.67 ± 3.81%, 38.73 ± 1.50%, respectively (Figure 4C), and for F-MSN-DOX@RBC were 67.17 ± 1.95%, 53.73 ± 2.74%, 46.20 ± 3.75%, 26.50 ± 1.08%, respectively (Figure 4D). In comparison, the phagocytosis of F-MSN-DOX@RBC NPs by RAW 264.7 was found to be lower than that of F-MSN-DOX at the corresponding time point (i.e., 2, 6, 12, or 24 h), and the difference was statistically significant (*p* < 0.05), indicating that the erythrocyte membrane wrapping around F-MSN could effectively reduce the phagocytosis of NPs by macrophages and therefore might prolong the circulation time of NPs in vivo.

### 3.4. In Vivo Biodistribution

**Figure 5:**
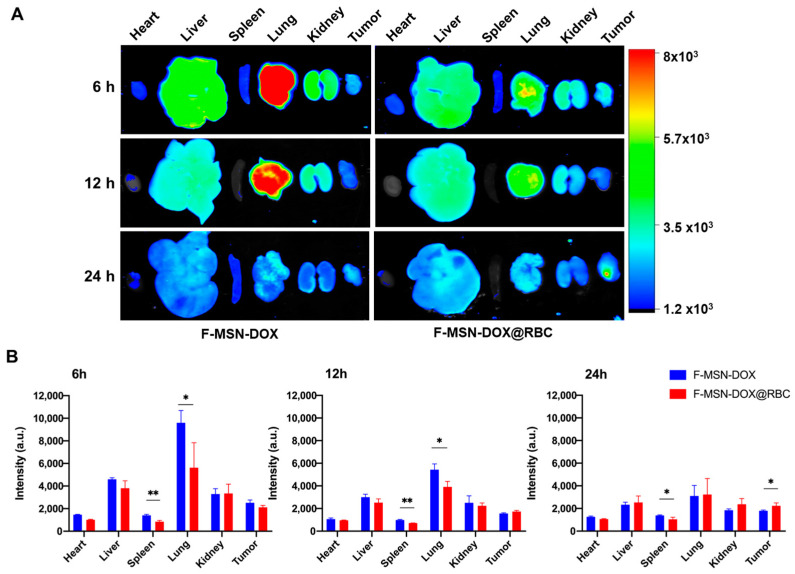
In vivo biodistribution of F-MSN-DOX and F-MSN-DOX@RBC. (**A**) Representative images of major organs. (**B**) Fluorescence quantification results at varied times. ** *p* < 0.01, * *p* < 0.05, n = 6.

We collected the major organs (hearts, livers, spleens, lungs, kidneys) and tumors to study the biodistribution of the two kinds of NPs. As shown in Figure 5A, the accumulation of F-MSN-DOX@RBC in the spleen and lung was much less than that of F-MSN-DOX, and the accumulation of F-MSN-DOX@RBC in the tumor was more significant than that of F-MSN-DOX at 24 h. This could be attributed to the fact that RBC membrane wrapping can effectively avoid the phagocytosis of NPs by the RES/MPS system, thus effectively reducing the accumulation of NPs in the lungs and spleens and allowing more NPs to enter the blood circulation, thus enhancing the accumulation of NPs in tumor tissue. It might also be that F-MSNs, without the wrapping of the RBC membrane, have a strong hydrophobicity and tend to agglomerate when injected into the body and, therefore, accumulated more in the lungs. 

### 3.5. In Vivo Anti-Tumor Efficacy

**Figure 6:**
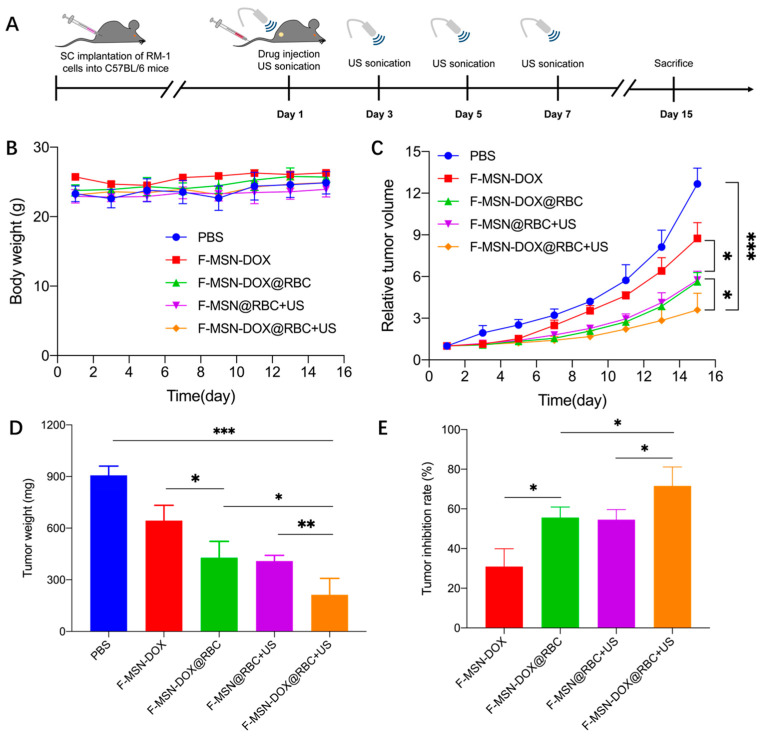
Anti-tumor efficacy assessment. (**A**) The anti-tumor experimental flow chart. (**B**) The body weight changes of different groups. (**C**) The relative tumor volume tracing. (**D**) The tumor weight with different treatments. (**E**) The RM-1 tumor inhabitation rate. *** *p* < 0.001, ** *p* < 0.01, * *p* < 0.05, (n = 6).

To assess the treatment efficacy in vivo, RM-1 cells were implanted subcutaneously in the right leg of C57BL/6 mice, and the first day was counted as when the tumor volume reached 80 mm^3^. All groups were injected with corresponding samples on the first day, with the US stimulus administered on days 1, 3, 5, and 7, respectively, and the mice were executed on day 15. The body weight and tumor volume changes were monitored every other day (Figure 6A). As shown in Figure 6B, there was no significant change in the body weight of the mice during treatment in each group, and there was no significant difference between groups, indicating that the F-MSN NPs, as well as the administered US, were safe. The relative tumor volume change in the F-MSN-DOX@RBC+US group was significantly smaller than in all other groups. Meanwhile, we found that the relative tumor volume in the F-MSN-DOX@RBC group was smaller than that in the F-MSN-DOX group, which indicated that the RBC membrane wrapping could improve the accumulation of NPs in the body, thus inhibiting tumor growth.

Furthermore, we found that US could effectively inhibit tumor growth by comparing the F-MSN-DOX@RBC+US group and the F-MSN-DOX@RBC group (Figure 6C). We stripped and weighed the tumor tissue, and the change in tumor weight was consistent with the change in relative tumor volume in each group (Figure 6D). The tumor inhibition rate in the F-MSN-DOX@RBC+US group was significantly higher than that of all other groups, indicating that a single intravenous drug injection with multiple administrations of US could effectively inhibit tumor growth (Figure 6E).

**Figure 7:**
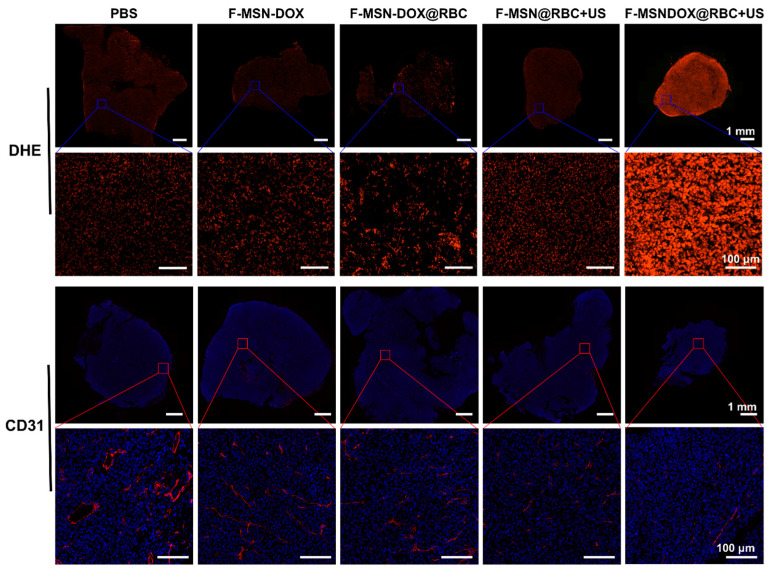
DHE and CD31 immunofluorescence staining of the ROS and blood vessels of RM-1 tumor. The scale bar is 1 mm in the upper row and 100 μm in the lower row.

To evaluate the anti-tumor angiogenic effects and ROS profiles in vivo, we used DHE and CD31 immunofluorescence staining. The red fluorescence in Figure 7 was significantly more intense in the F-MSN-DOX@RBC+US group than in any of the other groups, indicating a high ROS content. Our study revealed that the red fluorescence of the two groups of F-MSN@RBC+US and F-MSN-DOX@RBC was similar, which was explained by the fact that DOX can produce ROS on its own. This explains why the red fluorescence of the F-MSN-DOX@RBC+US group was significantly better than that of the F-MSN@RBC+US group. Cancer angiogenesis was assessed by platelet–endothelial cell adhesion molecule-1 (CD31). Moreover, we found that the F-MSN-DOX@RBC+US group could effectively inhibit tumor angiogenesis compared to other groups (Figure 7). 

**Figure 8:**
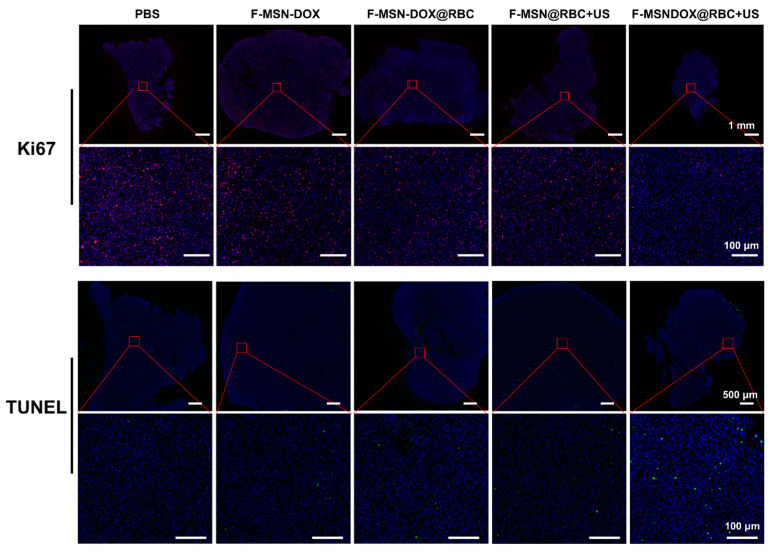
Ki67 and TUNEL immunofluorescence staining of the proliferation and apoptosis of tumor cells. The scale bar is 1 mm for Ki67 and 500 μm for TUNEL in the upper row, and all the scale bars in the lower row are 100 μm.

To determine the proliferation and apoptosis of tumor cells, we used Ki67 and TUNEL immunofluorescence staining, respectively. As expected, the F-MSN-DOX@RBC+US group had the least cell proliferation in the tumors (Figure 8). In Figure 8, the F-MSN-DOX@RBC+US group caused the most apoptosis of tumor cells among all the groups. By combining anti-angiogenesis, sonodynamic, and chemical therapy, a single injection of F-MSN-DOX@RBC could effectively inhibit tumor cell proliferation and promote tumor cell apoptosis.

**Figure 9:**
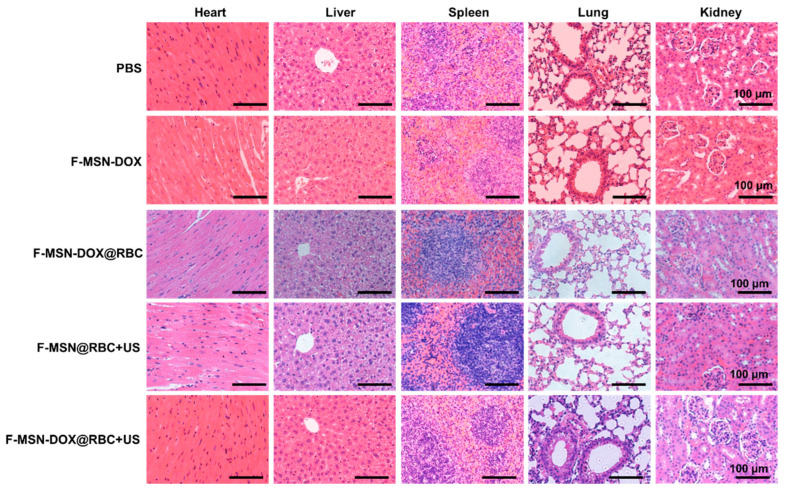
The HE staining histological images of the major organs under different treatments. The scale bar is 100 μm.

The safety of each group in vivo was further assessed by histopathological analyses of major organs. As shown in Figure 9, histopathological assessments were performed on the heart, liver, spleen, lung, and kidney. All in vivo treatment strategies did not result in significant histopathological damage, indicating their safety.

## 4. Discussion

In this study, we constructed erythrocyte membrane encapsulated biomimetic superhydrophobic drug-loaded mesoporous silica NPs, F-MSN-DOX@RBC, with uniform size, good stability, and high drug loading efficiency, which effectively reduced drug leakage. Erythrocyte membrane encapsulation greatly reduced the phagocytosis of the NPs by the monocytic macrophage system and increased the accumulation of NPs at the tumor site. Under US stimulus, F-MSN-DOX@RBC can generate cavitation, promote drug penetration into the tumor, reduce tumor angiogenesis, and effectively inhibit tumor growth without obvious toxic and side effects.

### 4.1. Ultrasound-Responsive NPs for Sonodynamic Therapy

Sonodynamic therapy has been recognized as a promising cancer treatment modality for its deep penetration in vivo in comparison to other modalities. Nanosonosensitizers, as a novel class of efficient sonosensitizer, have drawn more and more attention for their merits in comparison to other organic sonosensitizers. Osminkina et al. utilized porous silicon NPs coated by dextran as efficient sensitizers of SDT [50]. In addition, piezoelectric materials such as tetragonal BaTiO_3_ and black phosphorus have gradually attracted more and more attention [51,52]. When exposed to US, the nanopiezoelectric materials produced ROS and caused damage to tumor cells in an oxygen-free biological environment. In contrast to the above nanosonosensitizers, our superhydrophobic silica is another kind of sonosensitizer based on gas cavitation nuclei with a traditional cavitation mechanism. The merit of our sonosensitizer is that our silica was biodegradable for in vivo application. Studies have shown that when hydrophobic materials are placed in water, the lifetime of INBs formed by gas adsorption on the hydrophobic surface can reach several days or even weeks [27,53]. In addition to INBs on a bulk hydrophobic surface, INBs may also be stabilized on the surface of a solid NP. NPs would be more flexible and promising for various biomedical applications, provided that there were INBs or gas layers on their surface. These INBs or air layers might serve as cavitation nuclei for US ablations and US stimulation to modulate drug delivery. However, the number of studies that have started to apply INBs to develop US-responsive NPs is still limited.

Kwan et al. have shown that well-defined nanocups with distinct cavity sizes of 180, 260, and 600 nm in diameter were able to trap nanobubbles [30]. Upon exposure to US, the bubbles trapped within these NPs expanded and ejected a cavitating bubble [54]. In addition, these cavitation bubbles rapidly expanded and collapsed, emitting a broadband signal indicative of IC. The INBs trapped in nanocups have been demonstrated to promote drug and virus delivery in both in vivo and in vitro experiments [29,55]. Under US, these NPs can exclusively emit broadband emissions, which can be received by using a linear array transducer and reconstructed into passive acoustic maps to tell the cavitation position [29]. However, these nanocups were made of polystyrene, which is not biodegradable. Besides nanocups, Goodwin’s group has shown that porous silica NPs could be used for diagnostic US as a contrast agent since the silica NPs trapped gas within its pores. When exposed to HIFU (acoustic pressure as high as 9.4 MPa), gas within the pores nucleated cavitation bubble clouds and served as a contrast agent, and they explained the reason for this is due to the carbon impurities [25,32,33]. In another study by Zhu’s group, they designed hydrophobic mesoporous silica NPs or core–shell silica NPs and applied them as sonosensitizers for sonodynamic therapy under the excitation of continuous low-intensity US, and showed a significant antitumor effect [56,57]. Similarly, they also attributed these effects to the cavitation activities by combining US with the gas nuclei trapped in mesopores of NPs.

### 4.2. Improved Tumor Accumulation by Decoration of NPs

The immune system works to remove NPs, so long-term blood circulation is the key to promoting the accumulation of NPs in tumors and thus obtaining better therapeutic effects [58]. Therefore, researchers have focused on evading immune recognition and improving blood circulation time in vivo by developing NPs with external decorations, such as the classical PEG [42], poly-N-(2-hydroxypropyl)methacrylamide [59], poly-L-glutamic acid [60], zwitterionic polymers etc. [61]. Recently, the development of cell membrane-derived biomimetic nanoplatforms to facilitate the application of nanomedicine in biomedicine has attracted increasing attention [44,45,62,63,64]. NPs encapsulated by RBC membranes have a longer blood circulation time compared with ordinary NPs [65], which is due to the fact that the RBC membrane disguises the NPs as “self”, thus bypassing the immune recognition process. RBC membrane-encapsulated NPs rely on the “don’t eat me” signaling marker on the surface of RBCs, CD47, which evades immune clearance by binding to the signal regulatory protein-α (SIRP-α) receptor, thereby prolonging the circulation of NPs in vivo [47].

### 4.3. Anti-Vascular Therapy

Tumor vasculature transports oxygen and nutrients to supply the tumor tissue growth. Due to the rapid proliferation of tumor cells, the morphology of tumor vessels is leaky and fragile with a tortuous structure. Therefore, using an anti-vascular treatment, tumors are deprived of oxygen and nutrients as a result of disrupted blood vessels and reduced blood flow [66]. During chemical anti-vascular therapy, vascular disrupting agents dissolve the cytoskeleton of immature vascular endothelial cells. Nevertheless, some fragile or injured normal vessels may also be disrupted and experience adverse consequences as a result [67]. An US targeted microbubble destruction technique can apply physical anti-vascular therapy by local disruption of tumor vessels by IC [68]. The UTMD could also be guided by US imaging to prevent damage to the normal muscle or skin. Ho et al. have shown that vascular destruction could be induced by the so-called ADV process, with phase-shift droplet cavitation and IC by INBs on superhydrophobic silica NPs [16]. The vascular disruption can concurrently inhibit tumor growth with chemotherapy and sonodynamic therapy [16]. Our results have further justified the above conclusion.

### 4.4. In Vivo Monitorring of F-MSN

It is important to monitor the biodistribution of F-MSN since the biodegradablity of the silica NPs is still debated. It is easy to load F-MSN with gadolinium for MRI monitoring or iodine for CT imaging. Interestingly, the fluorinated F-MSN was able to be used as an F-19 MRI contrast agent for in vivo biodistribution monitoring with high sensitivity.

### 4.5. Study Limitation

Our study utilized erythrocyte membranes for modification, to improve the dispersion of superhydrophobic nanomaterials and their biological distribution. However, erythrocyte membranes do not have the ability to actively target themselves, and their targeting ability is not outstanding compared to other materials. Active-targeting strategies, including anti-vascular endothelial growth factor antibody and homologous targeting of tumor cell membrane coating, would further enhance the accumulation of F-MSNs in a tumor, enhancing the treatment efficiency. In addition, although the ability of mesoporous silica modified with fluorocarbon chains to adsorb interfacial bubbles to produce US responsiveness has been demonstrated in other literature, erythrocyte-modified superhydrophobic mesoporous silica produced excellent anti-tumor effects in vivo; however, whether the erythrocyte membrane modification affected the superhydrophobic properties of the nanomaterials and thus the adsorption of interfacial bubbles and US responsiveness was not explored in this study. Thirdly, a subcutaneous graft tumor was used as a tumor model, and it may need other imaging guidance to precisely locate orthotopic deep tumors. US has the merits of deep penetration and could be focused on a deep tumor under US and MRI imaging, which have been used as guidance modalities.

## 5. Conclusions

In summary, we successfully constructed erythrocyte membrane encapsulated biomimetic superhydrophobic drug-loaded mesoporous silica NPs, F-MSN-DOX@RBC, with uniform size, stable properties, and high drug loading efficiency, which effectively reduced drug leakage. Erythrocyte membrane encapsulation can reduce the phagocytosis of NPs by the monocytic macrophage system, and increase the accumulation of NPs at the tumor site. US combined with F-MSN-DOX@RBC can promote drug release, break the tumor blood vessels, and effectively inhibit tumor growth without obvious toxic and side effects.

## Figures and Tables

**Table 1:** Size distribution, concentration, and Zeta potentials of NPs.

Sample Name	Mean Size (nm)	Mean Concentration (Particles/mL)	Mean Zeta Potential (mV)
MSN	153.7 ± 86.2	1.62 × 10^10^ ± 2.98 × 10^8^	−17.97 ± 0.60
F-MSN	171.1 ± 53.9	1.44 × 10^10^ ± 2.42 × 10^8^	−19.03 ± 0.62
F-MSN-DOX	211.2 ± 72.4	8.37 × 10^9^ ± 1.16 × 10^8^	−21.70 ± 3.18
F-MSN-DOX@RBC	232.6 ± 78.8	8.19 × 10^9^ ± 2.30 × 10^8^	−35.57 ± 0.74

## Data Availability

Not applicable.
